# Global Genomic Surveillance of Middle East Respiratory Syndrome Coronavirus

**DOI:** 10.3201/eid3211.260668

**Published:** 2026-11

**Authors:** James Richard Otieno, Ahmed Mohammed Albarraq, Francis Amirtharaj Selvaraj, Amna Omair Al Mehairi, Esam Ibraheem Azhar, Christopher Ruis, Hasan Zackaria Ali Ishaq, Asma Abi Mohamed Shah, Zelalem Tadesse, Qiu Yu, Ihab El Masry, Hala Abou El Naja, Belinda L. Herring, Amal Barakat, Maria Van Kerkhove, Sophie von Dobschuetz, Lorenzo Subissi

**Affiliations:** World Health Organization, Geneva, Switzerland (J.R. Otieno, M. Van Kerkhove, S. von Dobschuetz, L. Subissi); Public Health Authority, Riyadh, Saudi Arabia (A.M. Albarraq); PureLab, Sheikh Khalifa Medical City, Abu Dhabi, United Arab Emirates (F.A. Selvaraj, A.O. Al Mehairi); Khalifa University, Abu Dhabi (F.A. Selvaraj); King Fahd Medical Research Center and Department of Medical Laboratory Sciences, King Abdulaziz University, Jeddah, Saudi Arabia (E.I. Azhar); University of Cambridge, Cambridge, UK (C. Ruis); Abu Dhabi Agriculture and Food Safety Authority, Abu Dhabi (H.Z.A. Ishaq, A.A.M. Shah); Food and Agriculture Organization of the United Nations, Rome, Italy (Z. Tadesse, Q. Yu, I. El Masry); World Health Organization, Cairo, Egypt (H.A. El Naja, A. Barakat); World Health Organization, Manila, the Philippines (B.L. Herring)

**Keywords:** Middle East Respiratory Syndrome Coronavirus, MERS-CoV, genomic surveillance, sequencing, One Health, camel trade, zoonotic surveillance, camels, zoonoses, viruses, respiratory infections

## Abstract

Middle East respiratory syndrome coronavirus (MERS-CoV) is a persistent zoonotic and epidemic threat. We analyzed 807 publicly available human- and camel-derived MERS-CoV genomes collected during 2012–2024 to assess global genomic surveillance. Most sequences originated from Saudi Arabia and the United Arab Emirates. Major gaps in surveillance persist across Africa and South and Central Asia despite large camel populations, active trade, and evidence of viral circulation. Phylogenetic analysis confirmed predominance of clade B in the Arabian Peninsula and clade C in East Africa, consistent with regional patterns. We identified uneven sampling, incomplete metadata, and long delays between sample collection and public sequence submission, often spanning years. Those limitations reduce the value of genomic data for timely situational awareness. Integrating genomic with population and trade data supports timelier, more representative, and coordinated One Health surveillance for MERS-CoV but reveals major blind spots.

Middle East respiratory syndrome coronavirus (MERS-CoV) was first detected in humans in 2012 (*1*). Saudi Arabia has recorded 84% of the 2,617 laboratory-confirmed human cases reported to the World Health Organization (WHO) as of May 2025 (*2*). Although limited human-to-human transmission has been documented within healthcare settings and in communities, most human infections are linked directly or indirectly to contact with infected dromedary camels, the main animal reservoir (*3*). In camels, infection is usually asymptomatic or mild (*4*), whereas infected humans can suffer severe respiratory illness and a crude case-fatality ratio of ≈37% (*5*). More than a decade after its discovery, MERS-CoV remains a public health concern because of its epidemic and pandemic potential, severe clinical outcomes, and the absence of licensed vaccines or therapeutics (*6*).

MERS-CoV belongs to the *Merbecovirus* subgenus within the genus *Betacoronavirus*, which also includes severe acute respiratory syndrome coronavirus (SARS-CoV), which caused the SARS epidemic in 2002–2003, and SARS-CoV-2, responsible for the COVID-19 pandemic (*7*). Three major clades within the MERS-CoV phylogeny have been identified, clades A, B and C, which differ in their geographic distribution and ecology. Differences in the clades phenotypic properties are still being investigated (*8*). Understanding MERS-CoV genetic diversity therefore remains necessary for situational awareness, interpretation of transmission patterns, and future preparedness planning (*9*,*10*).

Genomic epidemiology has become a critical component of pathogen surveillance, enabling the description of pathogen diversity, the contextualization of outbreaks, and identifying changes in circulating lineages over time (*11*). However, the utility of genomic surveillance depends not only on the existence of and access to sequence data but also on how representative, timely, and well annotated those data are. Recent reviews have documented major MERS-CoV surveillance and research gaps, such as incomplete virologic and serologic evidence in dromedary populations from Africa and South and Central Asia, uneven genomic characterization, and regional differences in research priorities (*12*–*14*). MERS-CoV sequence availability remains uneven across regions, and the extent to which currently available public genomic data support global situational awareness remains unclear.

MERS-CoV exhibits distinct epidemiologic patterns across regions. In Africa, clade C predominates in dromedary camels, yet we could not find evidence of officially reported autochthonous human cases despite serologic evidence of infection among occupationally exposed humans in some settings (*15*). Recent systematic reviews similarly highlight limited and uneven virologic and genomic evidence from camel populations in Africa (*12*,*13*). In the Arabian Peninsula, clade B predominates and is associated with high infection rates in dromedary camels and repeated spillover into humans, alongside occasional human-to-human transmission. Transmission intensity might vary seasonally, with increased spillover risk after the camel calving season, when maternally derived immunity in young camels declines and susceptibility increases (*16*). Although the reasons underlying those regional differences are not fully resolved, ex vivo studies suggest biological differences between clades, including variation in replication efficiency and host cell entry, that might contribute to their distinct epidemiologic patterns (*17*). Of note, recent detection of clade B sequences in camels from Africa suggests possible geographic expansion and underscores the importance of representative genomic surveillance (*18*,*19*).

We evaluated the current landscape of publicly available MERS-CoV genomic data as a surveillance resource. Rather than reconstructing the full evolutionary history of MERS-CoV, we assessed whether existing genomic data support timely and representative situational awareness. Specifically, we assessed sequence availability by country, host, and year; examined the completeness and operational usefulness of accompanying metadata; analyzed turnaround time between sample collection and public sequence submission; and contextualized genomic sampling against camel population size and reported camel trade flows. Our findings highlight practical gaps that might limit genomic risk assessment and suggest priorities for strengthening One Health surveillance.

## Methods

### Dataset Curation

On August 18, 2026, we retrieved all MERS-CoV sequences from GenBank by using the following taxonomy identifier query: (txid1335626[Organism:exp] OR txid1298362[Organism:exp] OR txid1306931[Organism:exp]) (Appendix 1) (*20*). We restricted analyses to human- and camel-derived sequences because these hosts represent the main epidemiologic interface for zoonotic transmission. To improve dataset reliability and comparability, we excluded sequences containing >10% ambiguous bases or <15,000 nucleotides, ≈50% of the reference genome length (30,119 bp) (National Center for Biotechnology Information RefSeq [https://www.ncbi.nlm.nih.gov/refseq] identification no. NC_019843.3). We supplemented missing or incomplete metadata, including geographic origin, host species, and collection dates, where possible through cross-referencing with published literature and public reports. We used the resulting curated dataset for all downstream analyses.

To support standardized genomic classification and quality assessment, we also developed a MERS-CoV Nextclade community dataset on the basis of the curated reference genome and clade framework used in this study (https://nextstrain.org/community/jrotieno/mers_cov). This resource is intended to enable more consistent clade assignment and interpretation across MERS-CoV genomic analyses.

### Sequence Alignments and Phylogenetic Analysis

We conducted multiple sequence alignment of the MERS-CoV genome sequences at the nucleotide level by using MAFFT version 7.526 with default parameters (*21*). We undertook manual inspection and editing of the alignments on AliView version 1.30 to ensure robust and accurate alignment of the dataset (*22*). We reconstructed phylogenetic trees by using IQ-TREE version 3.1.3 and by using the best-fit nucleotide substitution model (general time reversible plus empirical base frequencies plus invariable sites plus discrete gamma model with 4 rate categories) identified by using ModelFinder on the basis of the Bayesian information criterion (*23*,*24*). We used ultrafast bootstrap analysis (1,000 replicates) to assess branch support (*25*), and visualized trees in FigTree version 1.4.4 (https://tree.bio.ed.ac.uk/software/figtree).

We verified MERS-CoV clade assignments through comparison with reference sequences and analysis of tree topology. To validate clade assignment accuracy, we considered additional metadata, including geographic origin and host species.

Because this study evaluated public genomic data as a surveillance resource, our phylogenetic analyses contextualized known MERS-CoV clades across regions, hosts, and time rather than inferring detailed transmission histories or evolutionary rates. To summarize genetic diversity within clades, we calculated the mean and median pairwise distances by using the *dist.dna* function (model = raw) from the ape package in R version 4.6.1 (The R Project for Statistical Computing, https://www.r-project.org), applying pairwise deletion to handle missing data.

### Turnaround Time Analysis

To evaluate timeliness of public genomic data sharing, we calculated turnaround time (TAT) as the interval between sample collection and public sequence submission dates. We excluded sequences lacking complete collection or submission date metadata. We summarized TAT primarily by using median values to reduce the influence of extreme outliers and stratified results by WHO region, host, and year when possible. Because rapid sequence availability is central to public health value of genomic surveillance, we treated TAT as an operational indicator of surveillance performance rather than a virologic characteristic.

### Camel Population and Movement Data

To contextualize genomic sampling in relation to camel populations, trade patterns, and sequence sampling density, we used camel population and trade data from the Food and Agriculture Organization of the United Nations statistics database (FAOSTAT, https://www.fao.org/faostat). FAOSTAT reports aggregate camel populations without distinguishing between dromedary and Bactrian camels. We used data as broad contextual indicators of camel-associated surveillance coverage and not as species-specific estimates of dromedary reservoir size.

To align with the temporal coverage of the MERS-CoV sequence data, we restricted analyses to 2013–2024 for camel population and trade metrics. Camel data from 2025 were unavailable, and 2012 was excluded from some summary analyses because only 6 genomic sequences were available from that year.

We calculated annual camel sequence sampling density per 1 million camels and estimated country-level sampling density by using the median camel population over the study period. Those metrics were defined as (Figure 9):

**Figure 9:**
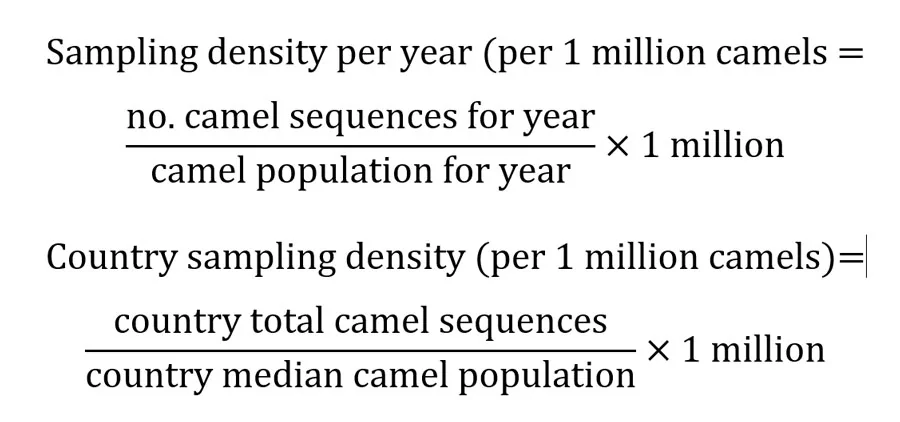
We calculated annual camel sequence sampling density per 1 million camels and estimated country-level sampling density by using the median camel population over the study period.

We visualized live camel trade flows to identify settings where genomic surveillance might be limited relative to the scale and connectivity of animal movement networks. Those analyses were intended to provide ecologic and operational context for surveillance interpretation, rather than to directly infer viral movement through trade.

## Results

### Geographic Concentration of MERS-CoV Genomic Data

We identified 1,738 MERS-CoV sequence records. After restricting the dataset to human- and camel-derived sequences and applying predefined length, ambiguity, and metadata curation criteria, we retained 807 sequences for analysis. Sequences represented 18 countries across 6 WHO regions, and collection dates spanned 2012–2025 (Appendix 1). The dataset comprised 449 (55.6%) camel-derived sequences and 358 (44.4%) human-derived sequences. Most sequences belonged to clade B (89.3%; n = 721 sequences, 356 human, 365 camel), whereas clade C accounted for 83 genomes (10.3%, all camel) and clade A for 3 genomes (0.4%, 2 human, 1 camel).

Of the 807 genomes, 52.5% originated from Saudi Arabia, making it by far the largest contributor to the public MERS-CoV sequence record (Figure 1). Together, Saudi Arabia and the United Arab Emirates (UAE) accounted for 75.0% of all genomes analyzed. Saudi Arabia contributed both camel- and human-derived sequences from many years, whereas the UAE dataset was concentrated during 2013–2017 and was predominantly camel-derived. The remaining countries contributed <5% of all genomes individually, highlighting the narrow geographic basis of the current public evidence base (*15*,*26*–*30*).

**Figure 1:**
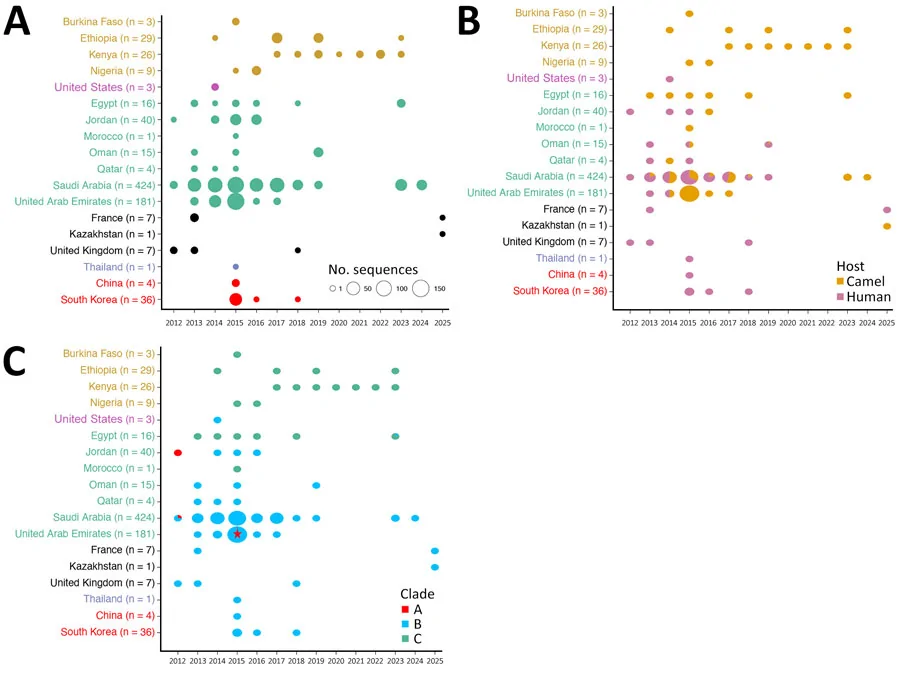
Breakdown of Middle East respiratory syndrome coronavirus sequences detected by country, host, and clade by year during global genomic surveillance, 2012–2025. A) Number of publicly available curated genome sequences by country and year of sampling. B) Proportion of sequences by host. C) Proportion of sequences by clade. The red star for United Arab Emirates in 2015 denotes a single clade A sequence. Available sequences by year: 2012, n = 8; 2013, n = 69; 2014, n = 115; 2015, n = 320; 2016, n = 75; 2017, n = 86; 2018, n = 20; 2019, n = 32; 2020, n = 1; 2021, n = 3; 2022, n = 7; 2023, n = 35; 2024, n = 15; 2025, n = 2. The total number of available sequences is shown in parentheses for each country in the y-axis. Countries are colored by World Health Organization region: gold, Africa Region; magenta, Region of the Americas; green, Eastern Mediterranean Region; black, European Region; blue, South-East Asia Region; red, Western Pacific Region.

Regionally, the WHO Eastern Mediterranean Region (EMR) (including Morocco, Tunisia, Libya, Egypt, Sudan, Djibouti, and Somalia) contributed 681 (84.4%) genomes to the public dataset. However, the WHO African Region (AFR) contributed only 67 (8.3%) genomes, indicating substantial underrepresentation of key transmission settings in the global genomic dataset. Sequences were also available from the WHO European region (n = 15, from France, the United Kingdom, and Kazakhstan) and the Region of the Americas (n = 3, from the United States). The human sequences from those 2 regions were mainly associated with travel-related MERS-CoV cases exposed in the Arabian Peninsula and rare, limited onward human-to-human transmission (*31*,*32*). The Kazakhstan sequence was obtained from a resident camel and characterized as clade B. We used WHO regional groupings to summarize surveillance contributions and operational patterns, not to imply biologically homogeneous evolutionary units.

TAT is a key determinant of the operational value of genomic surveillance (*33*). Across the global MERS-CoV dataset, TAT varied markedly by region and time (Appendix 2 Figure). Early submissions, particularly in 2013–2014, had shorter delays, whereas more recent submissions often reflected substantially longer lags, in some cases exceeding 5 years. Overall, median TAT increased over time, with wide variability between countries and submission periods. Our results indicate that MERS-CoV sequences might not be available in time to support real-time situational awareness, outbreak interpretation, or timely assessment of changes in circulating viral diversity.

### MERS-CoV Clades Phylogenetic Structure, Geographic Distribution, and Host Associations

MERS coronaviruses exhibit distinct evolutionary and epidemiologic characteristics by clade and host (Figures 2–4). Clade C (n = 78) viruses are characterized by long branch lengths (Figure 2) and a median pairwise nucleotide distance of 159 nucleotide bases (mean 152 bases, SD + 0.80), consistent with their genetic divergence within camel populations sampled in both WHO EMR and AFR (Figures 3, 4). Whereas clade A had too few genomic sequences (n = 3) to generate a meaningful estimate, clade B (n = 716) had a median pairwise distance of 57 bases (mean 68 bases, SD + 0.06).

**Figure 2:**
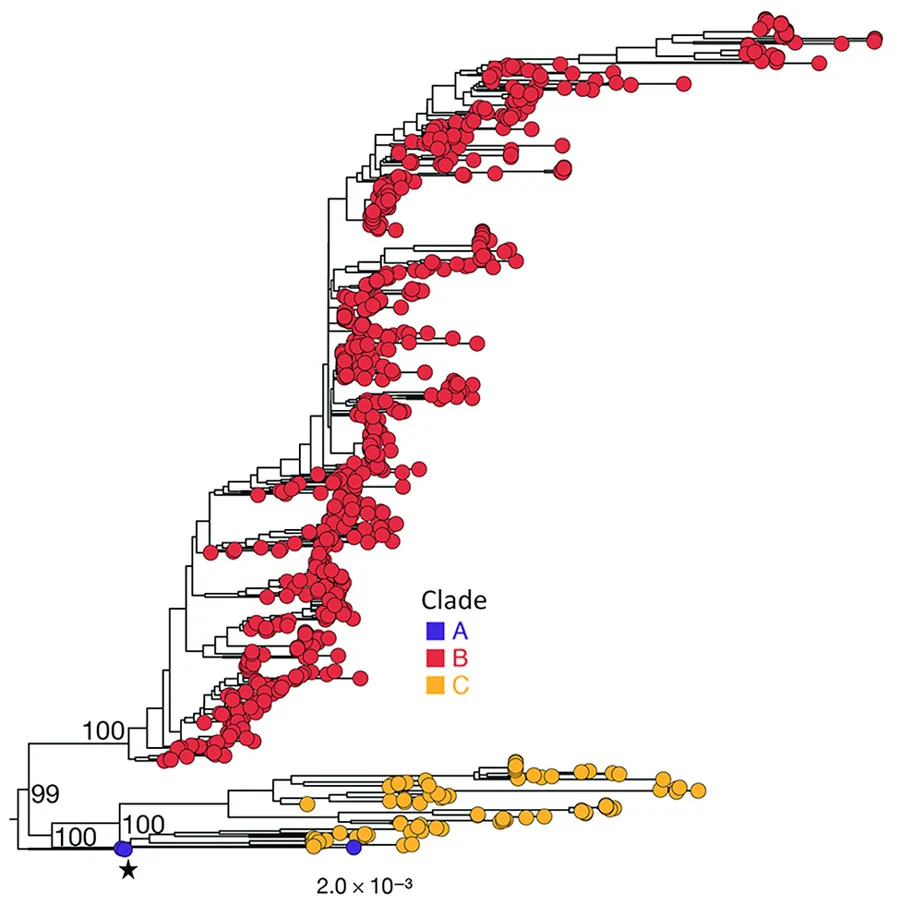
Maximum-likelihood phylogenetic tree highlighting the primary Middle East respiratory syndrome coronavirus clades described during global genomic surveillance, 2012–2025. The tree is rooted on a clade A sequence from GenBank (accession no. KC776174.1), indicated by the black star. The branches are colored by clade. Bootstrap values are shown for major nodes.

**Figure 4:**
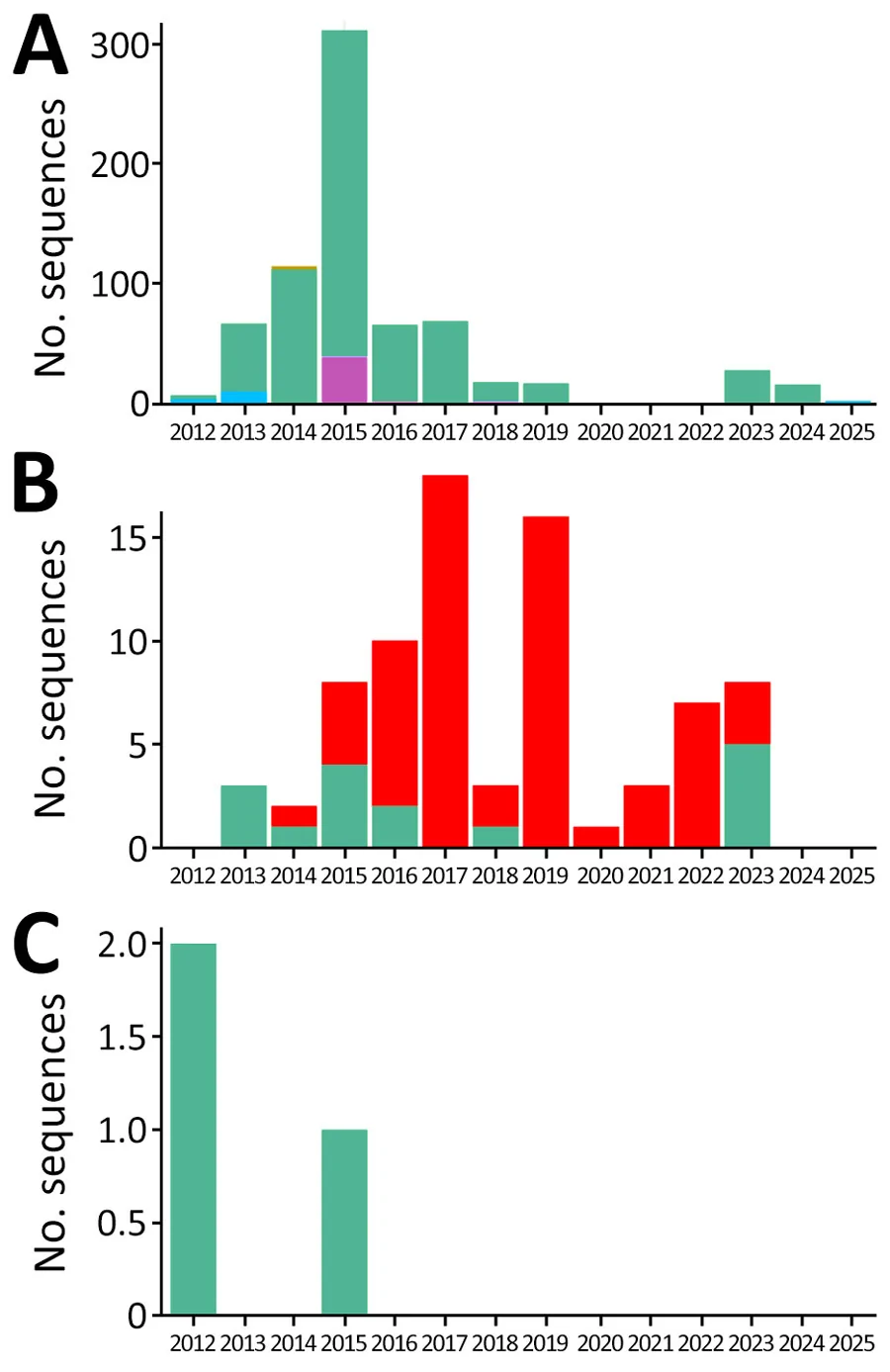
Temporal and regional distribution of Middle East respiratory syndrome coronavirus sequences for each clade described during global genomic surveillance, 2012–2025. A) Clade B, n = 721; B) clade C, n = 83; C) clade A, n = 3. Bar colors indicate World Health Organization region: gold, Africa Region (AFR); magenta, Region of the Americas (AMR); green, Eastern Mediterranean Region (EMR); black, European Region (EUR); blue, South-East Asia Region (SEAR); red, Western Pacific Region (WPR).

**Figure 3:**
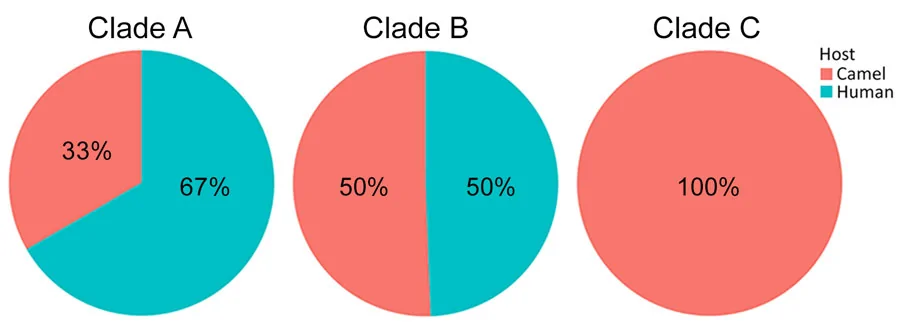
Percentages of clades A, B, and C Middle East respiratory syndrome coronavirus sequences, by host species, described during global genomic surveillance, 2012–2025.

In publicly available genomic data, in EMR, clade B has replaced clade A, with the last clade A sequence from the region collected from a camel in 2015 (*34*). Although clade B MERS-CoV sequences have historically been restricted to the Arabian Peninsula, recent genomic surveillance has documented the introduction of clade B sequences into the continent of Africa, specifically identified in dromedary camels from Egypt (*19*). As clade B continues to diversify (Figure 5, panel A), those viruses have been characterized into multiple lineages (*35*–*37*). Apart from a single travel-related human sequence collected in December 2025, the most recent human clade B sequences date back to January 2024, underscoring the need to monitor geographic distribution and lineage composition in exposed camel and human populations.

**Figure 5:**
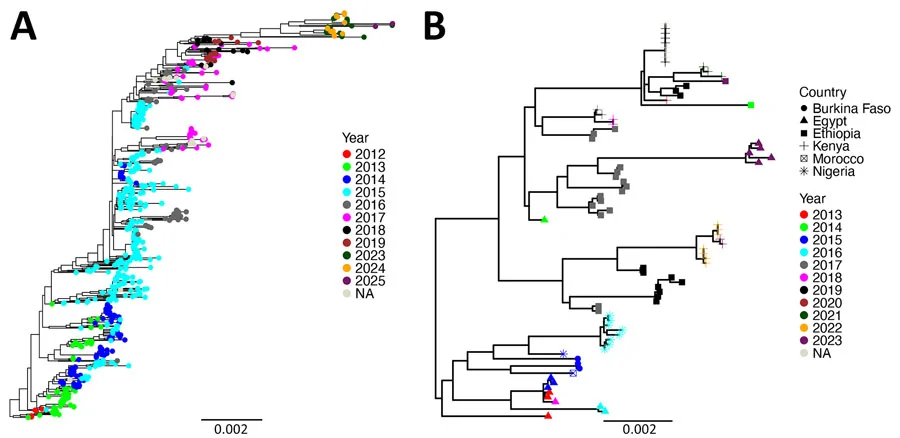
Maximum-likelihood phylogenetic trees of Middle East respiratory syndrome coronavirus described during global genomic surveillance, 2012–2025. A) Clade B sequences. The tips are colored by the year of sample collection. B) Clade C sequences. The tip-shape represents the country of isolation, and the color represents the year of sample collection. Scale bars indicate nucleotide substitutions per site.

Clade C has been identified in WHO AFR (Kenya, Burkina Faso, Ethiopia, and Nigeria) and EMR (Egypt and Morocco), with all the sequences coming from continental Africa (*29*,*38*,*39*). The clade C phylogenetic tree (Figure 5, panel B) demonstrates temporal and geographic clustering, especially among sequences from Ethiopia and Kenya, including recent clusters from Kenya during 2022–2023, compatible with ongoing circulation and diversification in parts of East Africa. Sequences from Egypt were found across multiple sampling years and included a recent 2023 camel-associated cluster, whereas sequences from Nigeria formed a geographically distinct but temporally limited cluster during 2015–2016 (*19*). Sparse and uneven sampling limits wider regional interpretation. To date, clade C has been associated with human infections only indirectly (*15*); however, underreporting cannot be ruled out (*17**)*. 

### Surveillance Gaps and Implications for MERS-CoV Transmission

Among 47 countries with available camel population data (Figure 6), 27 had median populations >100,000 animals and 9 had median populations >1 million animals. Because FAOSTAT does not distinguish dromedary from Bactrian camels, those figures represent aggregate camel populations and should be interpreted as contextual indicators rather than direct estimates of the distribution of the MERS-CoV dromedary reservoir. Camel-derived MERS-CoV genomes were available from 12 countries, but only 10 (37.0%) of the 27 countries with median camel populations of >100,000 animals were represented. That comparison indicates gaps in genomic surveillance relative to camel populations and recognizes that the denominator is not species-specific.

**Figure 6:**
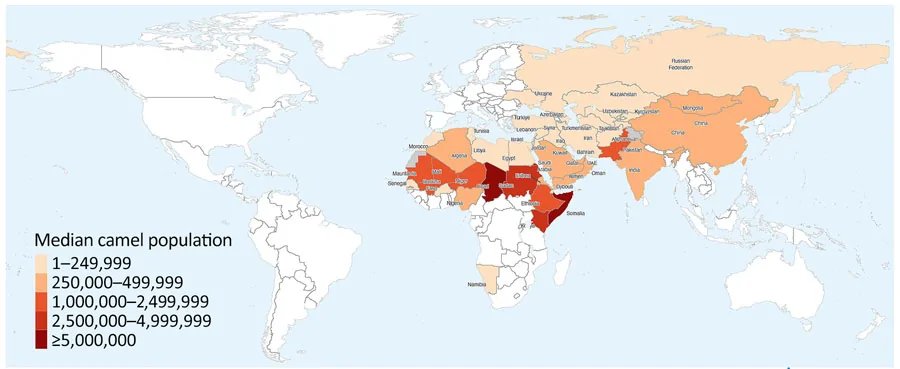
Median camel populations by country described during global genomic surveillance, 2012–2025. Map courtesy of the World Health Organization.

We calculated a contextual sampling-density metric as the number of genomes per 1 million camels, stratified by country and year (Figure 7). The data demonstrated disparities in sampling efforts; genomic sequences per 1 million camels were a median of 353 for Saudi Arabia, 334 for UAE, 103 for Egypt, 20 for Ethiopia, 10 for Qatar, and 7 for Kenya. No formal minimum sampling density is established according to our literature review; nevertheless, those findings demonstrate substantial heterogeneity and support more consistent, geographically representative camel surveillance.

**Figure 7:**
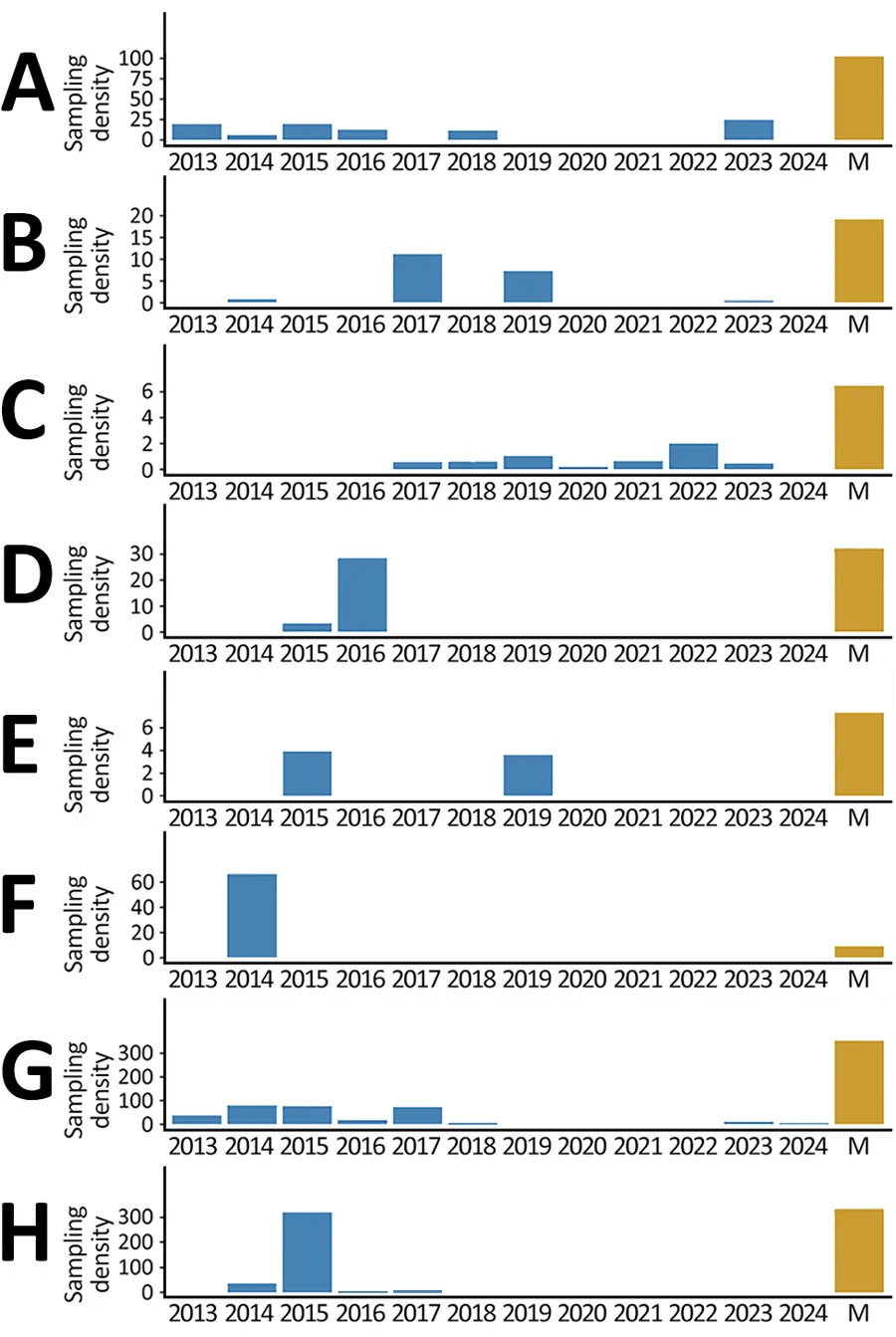
Middle East respiratory syndrome coronavirus sequence sampling density (sequences/1 million camels) by country and year described during global genomic surveillance, 2012–2025. A) Egypt; B) Ethiopia; C) Kenya; D) Nigeria; E) Oman; F) Qatar; G) Saudi Arabia; H) United Arab Emirates. The median sampling density per country is shown by the golden-brown bar to the right. y-axes differ among countries. The Food and Agriculture Organization of the United Nations statistics database does not distinguish dromedary from Bactrian camels; data are therefore presented as contextual indicators of camel-associated surveillance coverage and should not be interpreted as species-specific estimates of dromedary reservoir coverage.

Live camel trade might create opportunities for cross-border virus movement, but currently available trade and genomic data are insufficient to quantify that relationship directly. Nevertheless, the scale and complexity of camel trade flows highlight surveillance blind spots in several highly connected settings (Figure 8). We observed substantial trade activity among countries in EMR and between parts of Africa and the Arabian Peninsula, although genomic data remained absent or outdated for several countries participating in those networks. Those findings support stronger surveillance at transit, market, and other animal-human interface settings. Of note, trade flows are not evidence of MERS-CoV infection, infected-animal movement, or viral circulation; they are presented only as contextual indicators of animal-movement connectivity. Australia is a useful example because its large feral dromedary population might not be fully represented in conventional livestock statistics (*40*). The small Australia-directed flows shown (Figure 8), 5 camels in 2015 and 1 in 2021, indicate animal movement only and should not be interpreted as evidence of MERS-CoV infection or circulation in Australia.

**Figure 8:**
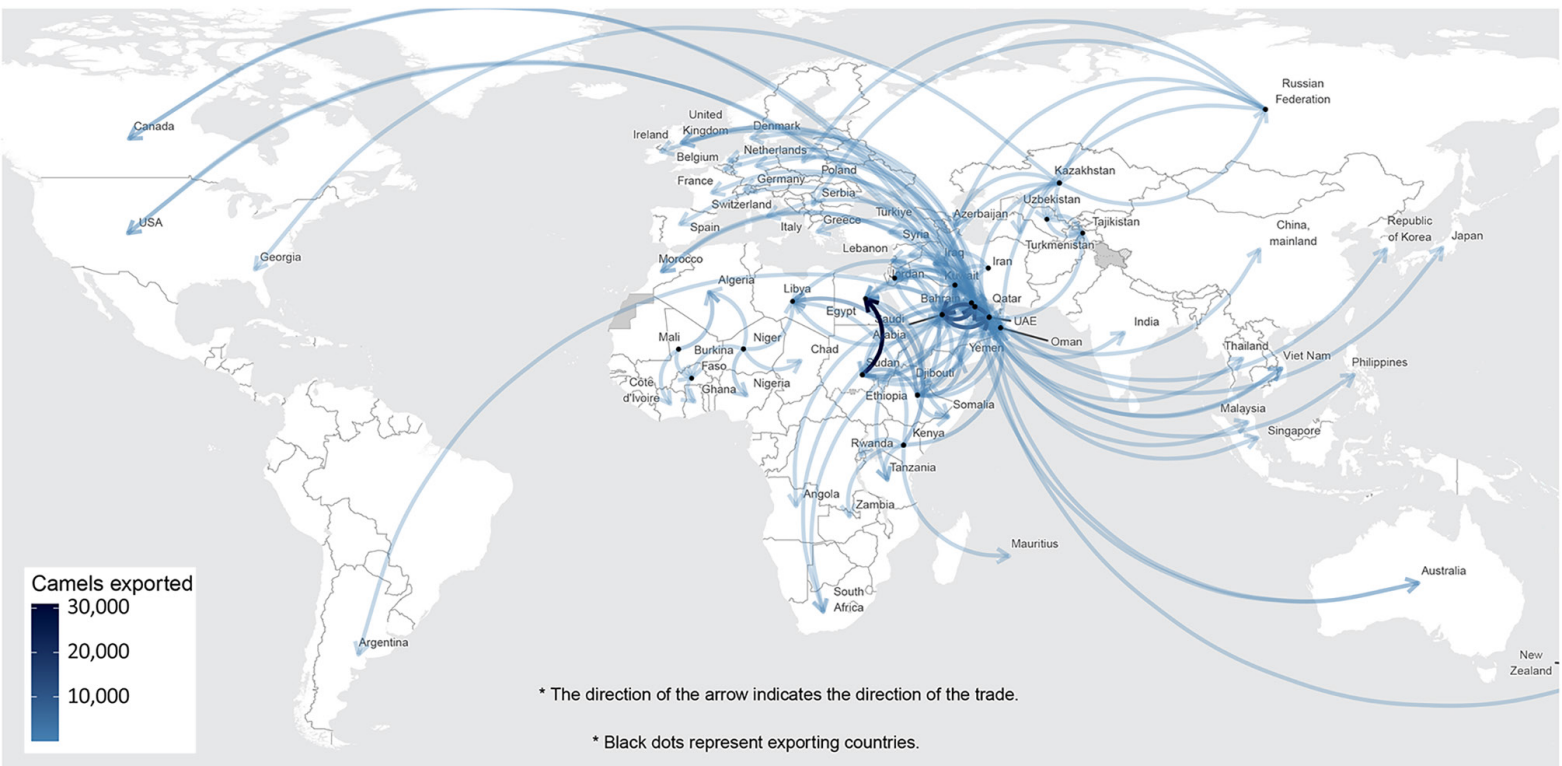
Live camel exports between countries described during global genomic surveillance, 2013–2024. The arrow tail represents the source country and the arrowhead represents destination country. The color of the arrow represents the number of camel exports. Trade flows are contextual indicators of animal movement connectivity and do not provide evidence of Middle East respiratory syndrome coronavirus infection, infected-animal movement, viral introduction, or viral circulation. The Australia-directed flows represent 6 animals in total, 5 in 2015 and 1 in 2021, and should be interpreted accordingly. Map provided by the World Health Organization Health Emergencies Programme using data from the Food and Agriculture Organization of the United Nations statistics database.

## Discussion

We evaluated the extent to which publicly available genomic data support global situational awareness for MERS-CoV. More than a decade after MERS-CoV was first identified, the public genomic record remains highly concentrated in a small number of countries, with major gaps in representativeness, metadata completeness, and timeliness. Those limitations constrain not only comparative phylogenetic interpretation but also the practical use of genomics for surveillance, risk assessment, and operational decision-making across both human and animal health systems.

Available genomic data show that clade B dominates among publicly available human and camel genomes from the Arabian Peninsula, whereas clade C remains largely represented by camel-derived genomes from Africa. Those findings are consistent with previous reports and support marked regional phylogenetic structure. However, the limited and uneven availability of genomes, particularly from settings in Africa and South and Central Asia, means that crucial components of viral diversity might remain unsampled. Recent MERS-CoV clade B genomes detected in camels originating from Egypt and Kazakhstan underscore the importance of geographically representative genomic surveillance and generation of full-length genome sequences for timely risk assessment (*18*,*19*). Although the current data identify surveillance gaps and broad phylogenetic structure, they do not enable definitive conclusions about global evolutionary dynamics.

Our findings complement recent reviews of MERS-CoV epidemiology, camel infection, genomic features, and regional research priorities (*12*,*13*). Whereas previous studies primarily synthesized epidemiologic or genomic evidence, our study evaluates the public genomic record as an operational surveillance resource by quantifying geographic representation, metadata completeness, submission delays, and coverage relative to camel populations and trade networks. We observed large disparities in camel genomic sampling density across countries with substantial camel populations. Median sequence density was far higher in Saudi Arabia and UAE than in Kenya or Ethiopia, whereas several countries with large camel populations lacked publicly available genomic data. Those disparities likely reflect targeted studies, differences in laboratory capacity, funding, and data-sharing practices. Our findings support context-appropriate reporting standards for camel genomic surveillance and the need for clearer denominator data.

The integration of camel population and trade data provides an operational perspective on where genomic surveillance might be most incomplete. Although our analyses do not directly demonstrate trade-mediated viral movement, they show that several countries participating in major trade networks have limited, outdated, or no public genomic data. That gap is particularly relevant for routes linking endemic areas and high-contact animal movement corridors. Better integration of genomic, epidemiologic, and animal movement data would improve interpretation of cross-border risk and help prioritize surveillance in high-connectivity settings.

A contribution of note is the analysis of TAT from sample collection to public sequence submission. Although sequence availability is often used as a proxy for surveillance effort, timeliness is equally critical for public health utility. Many MERS-CoV genomes are shared only after substantial delays, often years after collection, limiting their utility for timely risk assessment or outbreak interpretation. Those delays likely reflect differences in surveillance design, sequencing workflows, publication timelines, and data-sharing practices. For MERS-CoV, improving TAT might be crucial, as well as increasing the absolute number of publicly available genomes, and particularly relevant for a zoonotic pathogen with sporadic spillover and fragmented surveillance.

The main challenge for MERS-CoV genomics is ensuring that sequence data are representative, timely, and interpretable enough to support public health action. Strengthening routine genomic surveillance in camels and humans, improving metadata completeness, and reducing delays in public data sharing would increase the operational value of MERS-CoV genomics.

Whole-genome sequencing should be applied strategically rather than universally. Targeted sequencing might be sufficient when the objective is a defined diagnostic, antiviral resistance, or vaccine-relevant genomic region. However, representative full or near-complete genomes provide broader surveillance value by supporting clade and lineage assignment, detecting unexpected genetic changes, assessing recombination, placing sequences in an evolutionary context, and investigating unusual epidemiologic events. A tiered approach combining routine diagnostic testing with representative whole-genome sequencing through national or regional reference laboratories would be appropriate.

Existing global initiatives, such as the WHO Coronavirus Network, provide a platform for strengthening laboratory and analytical capacity, enabling data sharing, and promoting harmonized approaches to genomic surveillance (*41*). However, network representation and national sequencing capacity remain uneven across regions with substantial camel populations or epidemiologic relevance to MERS-CoV. In countries where national sequencing capacity is limited, referral of selected positive specimens to regional reference laboratories could enable representative and timely whole-genome sequencing. The institutional, logistic, and policy factors influencing national participation or specimen referral warrant further investigation.

The first limitation of this study is that the available public genomic dataset is affected by substantial temporal and geographic bias, with more than 75% of genomes originating from Saudi Arabia, the UAE, and neighboring countries in the Arabian Peninsula. Of consequence, the analyses should not be interpreted as a complete description of global MERS-CoV evolutionary dynamics, but rather as an assessment of the current public genomic evidence base and resulting surveillance gaps. 

Second, crucial metadata fields were missing for a proportion of sequences, including collection dates, host information, and subnational geographic detail. Those gaps reduced the number of genomes included in some analyses, particularly TAT, and limited more detailed interpretation of local evolutionary patterns.

Third, camel population and trade data were derived from FAOSTAT and reflect officially collected data only. FAOSTAT does not distinguish dromedary from Bactrian camels and might misrepresent camel populations relevant to MERS-CoV surveillance. Trade statistics might also miss informal or undocumented animal movements and contain no information on infection status. Of consequence, camel population, sampling density, and trade comparisons should be interpreted as contextual indicators rather than precise estimates of dromedary-reservoir surveillance coverage or direct evidence of viral movement. Australia provides an additional example because its large feral dromedary population might not be fully captured by conventional livestock statistics (*40*).

Finally, because this study was designed to evaluate the utility of genomic data for surveillance, we did not perform additional phylogeographic, recombination, or phenotypic analyses. Recombination is a major driver of coronavirus evolution with potential implications for lineage-specific phenotypes and has been explored extensively in previous studies (*37*,*42*–*45*). Such analyses remain a necessary avenue for future work as more representative and timely genomic data become available.

This study reveals that publicly available MERS-CoV genomic data provide a necessary but incomplete foundation for global surveillance. The major constraints include limited sequence generation, uneven representativeness, incomplete metadata, and prolonged delays in public data sharing. By evaluating sequence coverage, TAT, and surveillance context alongside camel population and trade indicators, we identify practical gaps that currently limit the public health utility of MERS-CoV genomics. Strengthening representative sampling, improving the timeliness of sequence sharing, and embedding genomic surveillance within a coordinated One Health framework would enhance the value of those data for risk assessment, preparedness, and response. Improving camel population and trade datasets should also be prioritized. Achieving a One Health framework will require sustained coordination across human, animal, and environmental health sectors, including collaboration among WHO, the Food and Agriculture Organization of the United Nations, the World Organisation for Animal Health, and regional surveillance networks. Such efforts are essential for developing more integrated, timely, and actionable genomic surveillance systems for MERS-CoV and future zoonotic threats.

Appendix 1 Additional information about global genomic surveillance of Middle East respiratory syndrome coronavirus.

Appendix 2 Additional figure detailing turnaround time between sample collection and public sequence submission for human MERS-CoV isolates.
