# Supplementary material for: Global Genomic Surveillance of Middle East Respiratory Syndrome Coronavirus
Source: Emerg Infect Dis. 2026 Nov;32(11):1816–26. doi: 10.3201/eid3211.260668 (PMC13715891; doi:10.3201/eid3211.260668)
Supplement: Supplementary file 1 [file 26-0668-Techapp-s2.pdf]

Article DOI: https://doi.org/10.3201/eid3211.260668

EID cannot ensure accessibility for supplementary materials supplied by authors. Readers who have difficulty accessing supplementary content should contact the authors for assistance.

Global Genomic Surveillance of Middle East Respiratory Syndrome Coronavirus

Appendix 2

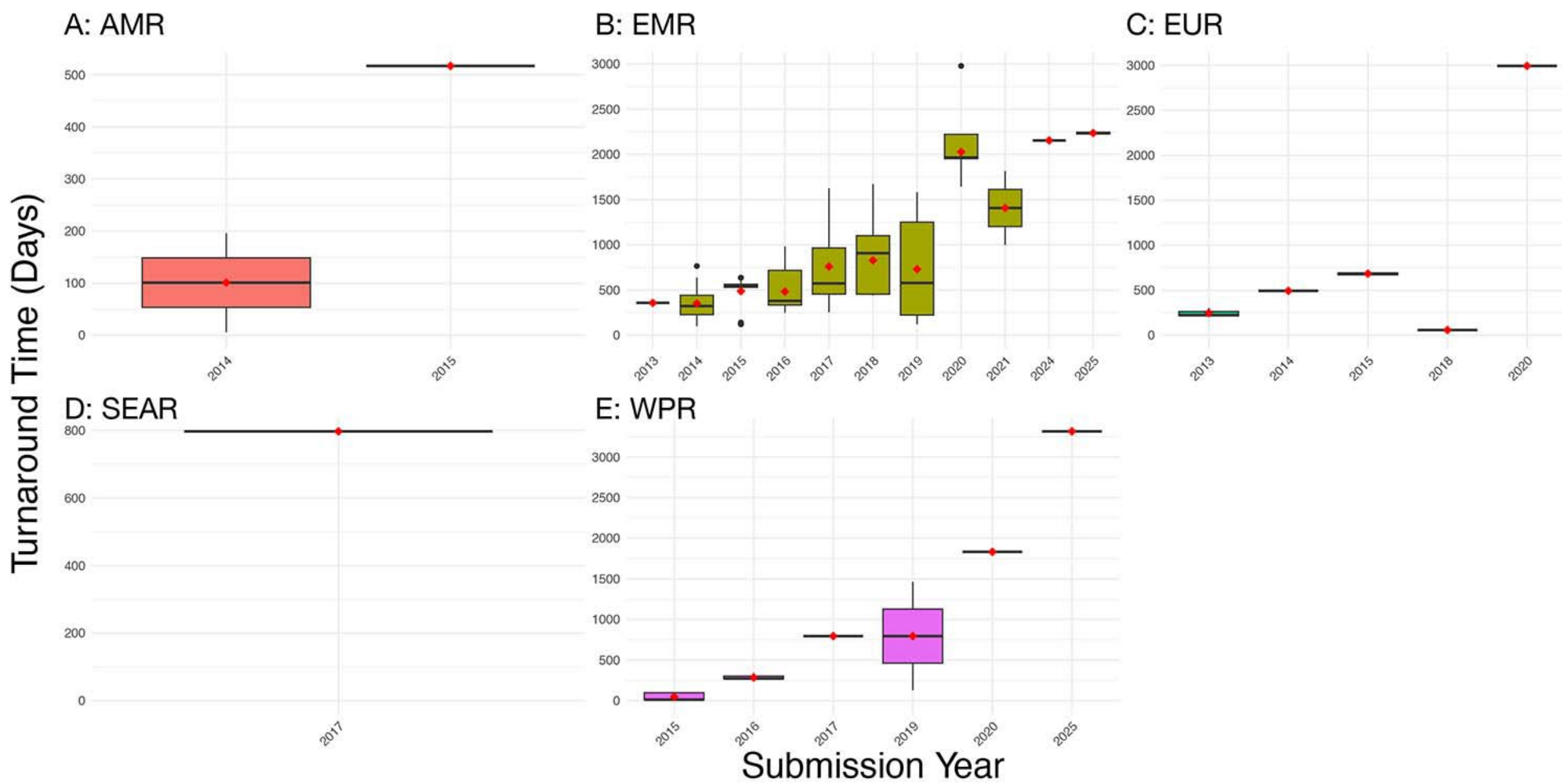

Appendix 2 Figure. Turnaround time (TOT) between sample collection and public sequence submission for human MERS-CoV isolates, stratified by WHO region and submission year. Boxplots summarize the distribution of TOT values (median, interquartile range, whiskers, and outliers); red diamonds indicate mean TOT. Panels show the WHO regions of the Americas (AMR), Eastern Mediterranean (EMR), Europe (EUR), South-East Asia (SEAR), and Western Pacific (WPR).
